# Automatic echocardiographic anomalies interpretation using a stacked residual-dense network model

**DOI:** 10.1186/s12859-023-05493-9

**Published:** 2023-09-27

**Authors:** Siti Nurmaini, Ade Iriani Sapitri, Bambang Tutuko, Muhammad Naufal Rachmatullah, Dian Palupi Rini, Annisa Darmawahyuni, Firdaus Firdaus, Satria Mandala, Ria Nova, Nuswil Bernolian

**Affiliations:** 1 Intelligent System Research Group, Faculty of Computer Science, Universitas Sriwijaya, Palembang, 30139 Indonesia; 2 Doctoral Program, Faculty of Engineering, Universitas Sriwijaya, Palembang, Indonesia; 3 Department of Informatic Engineering, Faculty of Computer Science, Universitas Sriwijaya, Palembang, Indonesia; 4 Human Centric Engineering, School of Computing, Telkom University, Bandung, Indonesia; 5 Division of Pediatric Cardiology, Department of Child Health, Mohammad Hoesin General Hospital, Palembang, Indonesia; 6 Division of Fetomaternal, Department of Obstetrics and Gynaecology, Mohammad Hoesin General Hospital, Palembang, Indonesia

**Keywords:** Cardiac septal defect, Classification, Deep learning, Echocardiography, Segmentation

## Abstract

Echocardiographic interpretation during the prenatal or postnatal period is important for diagnosing cardiac septal abnormalities. However, manual interpretation can be time consuming and subject to human error. Automatic segmentation of echocardiogram can support cardiologists in making an initial interpretation. However, such a process does not always provide straightforward information to make a complete interpretation. The segmentation process only identifies the region of cardiac septal abnormality, whereas complete interpretation should determine based on the position of defect. In this study, we proposed a stacked residual-dense network model to segment the entire region of cardiac and classifying their defect positions to generate automatic echocardiographic interpretation. We proposed the generalization model with incorporated two modalities: prenatal and postnatal echocardiography. To further evaluate the effectiveness of our model, its performance was verified by five cardiologists. We develop a pipeline process using 1345 echocardiograms for training data and 181 echocardiograms for unseen data from prospective patients acquired during standard clinical practice at Muhammad Hoesin General Hospital in Indonesia. As a result, the proposed model produced of 58.17% intersection over union (IoU), 75.75% dice similarity coefficient (DSC), and 76.36% mean average precision (mAP) for the validation data. Using unseen data, we achieved 42.39% IoU, 55.72% DSC, and 51.04% mAP. Further, the classification of defect positions using unseen data had approximately 92.27% accuracy, 94.33% specificity, and 92.05% sensitivity. Finally, our proposed model is validated with human expert with varying Kappa value. On average, these results hold promise of increasing suitability in clinical practice as a supporting diagnostic tool for establishing the diagnosis.

## Introduction

Congenital heart diseases (CHDs) are anatomical abnormalities of the heart and blood vessels that develop during the first trimester intrauterine pregnancy [1]. Most abnormalities do not exhibit symptoms of heart failure in utero because the placental circulation provides nutrients and oxygen to the growing fetus. However, after birth and the transition from fetal to newborn circulation, CHDs symptoms are marked by the closure of the fetal shunt, making the existence of this condition evident [2, 3] The incidence of CHDs in Asia is higher than the global average, reaching a ratio of 9.3 cases per 1000 live births [1]. However, in > 50% of newborns, CHDs are clinically undetectable during hospital discharge [4]. This condition accounts for 5% of all childhood deaths and 18% of deaths within the first year of life among liveborn infants [1, 2, 4].

Cardiac septal defects are one of the most common types of CHDs with varying levels from small to large. The three cardiac septal defects based on the defect position in the cardiac septum are atrial septal defect (ASD), ventricular septal defect (VSD), and atrioventricular septal defect (AVSD) [3]. Septal defects account for approximately 57.9% of CHDs cases worldwide [3]. Thus, cardiac septal defect detection has significant implications for pregnancy management. These conditions should be detected on routine ultrasonography at 18–22 weeks gestation [5]. However, from the 22-week gestation to the end of the 28^th^ week, the heart is no larger than 25 mm [5], and it is difficult to accurately identify the cardiac structure. If cardiac septal defects are not identified prenatally, they must be detected postnatally. However, sometimes they are not discovered until birth or even until the infant becomes an adult. Cardiac septal defects at birth can affect the structure and functionality of the heart. Signs and symptoms of severe cardiac defects often appear during the first few days, weeks, or months of a child’s life. A small defect may cause no problems, and in many cases, small defects heal on their own. Meanwhile, medium or large defects may require surgical repair in early life to prevent complications [6].

Early cardiac screening is crucial for the accurate prenatal and postnatal diagnosis of structural cardiac abnormalities [7]. The screening process utilizes complete planes of cardiac tissues to enable 90% detection of cardiac septal defects interpretation. Four planes are used prenatally in the analysis of cardiac structure and function prenatally: 4‐chamber, outflow tracts, and three‐vessel and trachea planes [6]. Five planes are used in postnatal cardiac structures: 4‐chamber, 5-chamber, subcoastal, parasternal long-axis, and parasternal short-axis planes. However, the 4-chamber plane is instrumental in delineating the fetus’s entire cardiac structure (i.e., the interatrial and interventricular septa). More than 60% of cardiac septal defects can be prenatally detected using the 4-chamber plane alone [8].

Accurate echocardiographic interpretation is complicated even for gynecologist fetomaternal and pediatric cardiology experts, due to the small size of the cardiac, imaging artifacts and speckle noise, fetal rib shadowing, missing boundaries, and similarity of anatomical structures [6, 7]. In addition, there is the incompatibility and non-uniformity of echocardiographic interpretation results by a physician. Even these discrepancies echocardiogram interpretation can occur between fetomaternal experts as well as pediatric cardiology experts [9]. A new artificial intelligence AI-based computer vision technology for assisted echocardiographic interpretation may be able to diagnose cardiac problems more quickly and accurately than a medical professional, significantly improving the odds of survival [10–12]. Such approach can identify subtle patterns and early signs of diseases that might be difficult for human eyes to detect.

AI and its subfield of deep learning (DL) with a convolutional neural network (CNN) architecture offer the prospect of descriptive echocardiographic analysis [11, 13–16]. CNN provide a new research opportunity to produce a precise and reliable method [17, 18]. They are distinguished by their ability to learn complex representations of raw data to improve the recognition of echocardiogram patterns [11, 13–15, 19]. Several automatic CNNs techniques for segmentation have been proposed to clearly recognize cardiac anatomical structures [18]. A dilated convolutional chain has been developed for the accurate segmentation of seven important fetal echocardiography anatomical structures in a 4-chamber plane [8].

Other studies have developed an accurate segmentation of cardiac septal defects using fetal echocardiography based on Mask-RCNN [11, 16]. However, the segmentation result of the proposed model cannot produce the final decision, and the result only segments the contour of the cardiac structure. This process produces an incomplete interpretation of the cardiac septal defect type, and hence, the post-processing stage should be added to consider the defect position when making a complete interpretation [20–22]. Therefore, the segmentation results from previous studies cannot provide a definitive finding on the type of cardiac septal defects.

Many studies have used a classification approach to determine the type of cardiac septal defects. A DL model with a CNN architecture successfully classified 15-standard planes of echocardiograms [12], and a CNN-based residual learning diagnostic system was developed for cardiac septal defects to improve diagnostic accuracy [15]. However, this approach is a black-box process and should be explained and visualized. The classification results without such a process are difficult to understand clinically. In addition, previous studies used only prenatal or postnatal echocardiograms separately; neither previous research used a combination of the two simultaneously. To support clinicians and make accurate and complete cardiac septal defect interpretation during prenatal and postnatal periods, the main contour of the cardiac septum should be segmented into normal and abnormal structures, features from segmented areas must be extracted, and the defect types should be automatically classified. Hence, it is desirable to develop a DL model with a CNN architecture for segmenting the cardiac anatomy and simultaneously classifying the defect position.

Even within seminal studies in the field, wide variations in design, methodology, and reporting remain, limiting the generalizability and applicability of the findings [11]. Thus, in this study, we propose a generalized DL model with a deep stacked CNN architecture based on instance segmentation and a classification approach for the automated echocardiographic interpretation of prenatal and postnatal data. To the best our knowledge, this is the first study to conduct such an experiment by incorporating these two modalities. The novelty and contributions of this study are as follows:

- A stacked residual-dense network model is designed for segmenting and classifying the cardiac septal defect to make an echocardiographic interpretation;
- The proposed model is generalized with incorporated prenatal and postnatal echocardiograms;
- The proposed model is evaluated using unseen data (new patient) and validated by five human-experts.

## Materials and methods

### Data preparation

Two modality devices were incorporated to produce an ultrasound video-recording dataset. Echocardiogram video were acquired from Muhammad Hoesin General Hospital, Palembang, Indonesia, during standard clinical practice between 2020 and 2022. The postnatal echocardiographic examination was recorded for a 5-s loop length using a Philips EPIQ 7C machine with X5-1 low-frequency imaging probes for small infants and 12S high-frequency imaging probes for infants, while the prenatal echocardiographic examination was recorded for a loop length of 10 s to 5 min using a GE Voluson E6 and Philips EPIQ 7C. The entire echocardiographic examination video was saved in digital imaging and communications in medicine (DICOM) format.

Video recording of echocardiogram examination for normal hearts and cardiac septal defects postnatally is performed with apical 4-chamber view, apical 5-chamber view, parasternal long axis view, parasternal short axis view, and subcostal view, whereas only apical 4-chamber view used for echocardiogram examination prenatally. Many types of cardiac septal defects, however, our study was only to predict certain types of cardiac septal defects due to limited data, namely ASD secundum, VSD perimembrane outlets and complete AVSD. For prenatal examination, the age of subjects ranged from 23 to 38 years old with the average of 30.8 years, with a gestation of 22–28 weeks (average 24.5 months), while for postnatal examination, the subjects were patients with cardiac septal defects aged 2 months–10 years (with average of 2.9 years). Table 1 presents the data distribution of the proposed model.

**Table 1:** Data distribution from prenatal and postnatal echocardiograms to evaluate the performance of the generated a stacked residual-dense network model on testing and unseen data

Learning process	ASD	AVSD	VSD	Normal	Total
Training	335	216	359	275	1185
Validation	49	20	37	54	160
Testing with unseen	44	38	45	54	181
Total					1526

### Data pre-processing

Pre-processing raw echocardiograms before feeding them to the network is an essential step for efficient training. The first aspect to consider is the existence of unexpected materials that may appear in the hearts of patients. The cardiac image should be annotated to perform quantitative analysis and reduce unexpected clinical parameters related to normal and abnormal conditions. The annotations is carried out by fetomaternal and pediatric cardiology experts. Our model focused only on cardiac defect objects, although all cardiac objects were annotated, including the atrial and ventricular regions. Based on the selected cardiac septal defect type, the data preparation is divided with two processes namely object annotation for segmentation and image labelling for classification.

#### Object annotation

**Fig. 1:**
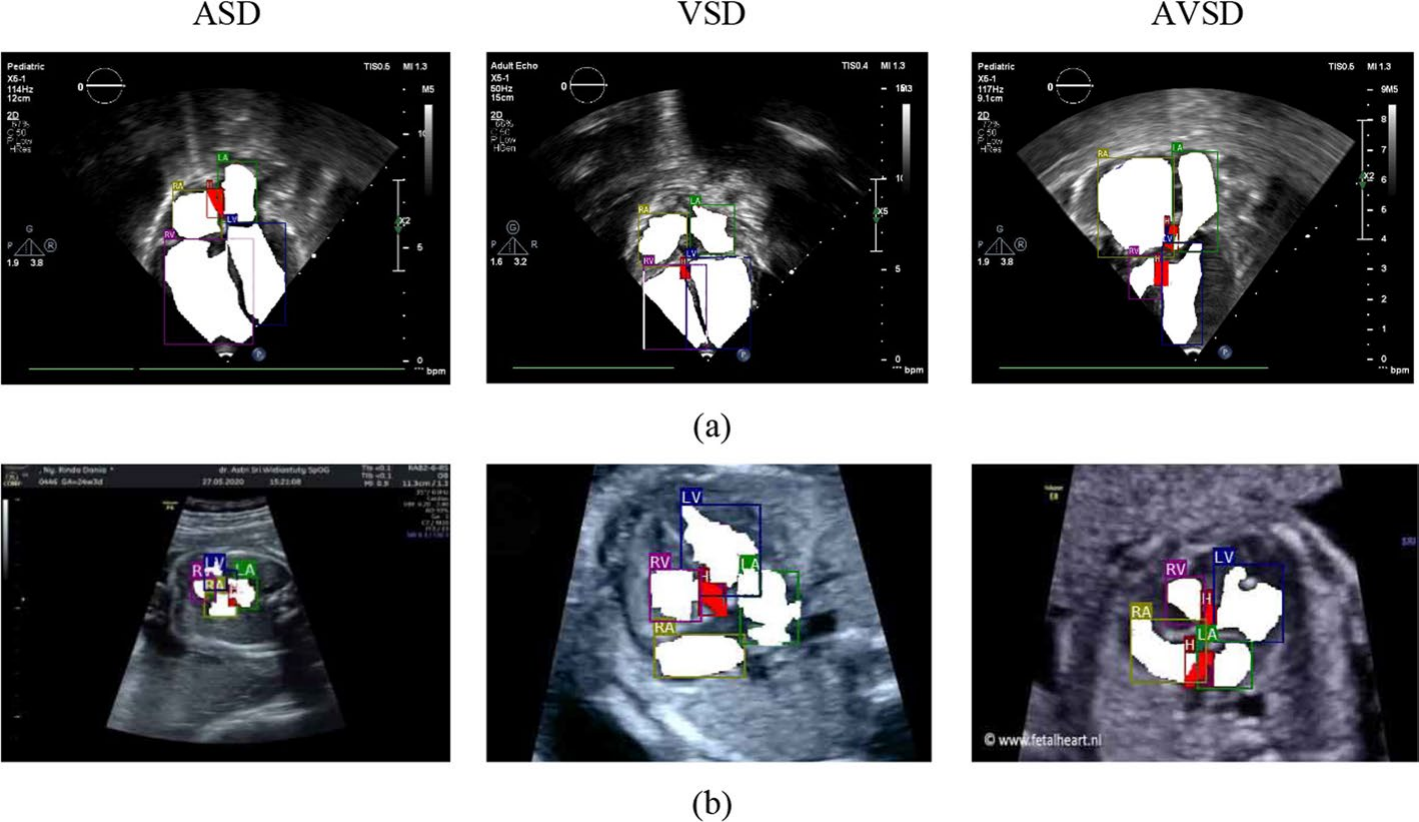
Example of the echocardiogram annotation process by experts to develop an AI model that can detect cardiac septal defects prenatally and postnatally. The cardiac chamber region is marked with white (right atrium [RA], left atrium [LA], right ventricle [RV], and left ventricle [LV]), whereas the defect area is marked with red. **a** Echocardiogram annotation of cardiac septal defect postnatally. **b** Echocardiogram annotation of cardiac septal defect prenatally

Segmentation is the process of automatic detection of boundaries within a 2D image. In the previous study, we have proposed the segmentation model for fetal echocardiography, and producing the satisfactory performance [19], such model architecture is improved based on two modalities for prenatal and postnatal echocardiogram. Object annotation is the process to mark the heart contours in the segmentation process (Fig. 1). In this process, video recording of echocardiogram examination on children’s heart is performed with apical 4-chamber view, apical 5-chamber view, parasternal long axis view, parasternal short axis view, and subcostal view. While echocardiogram examination on fetal’s heart only use apical 4-chamber view.

#### Images labelling

**Fig. 2:**
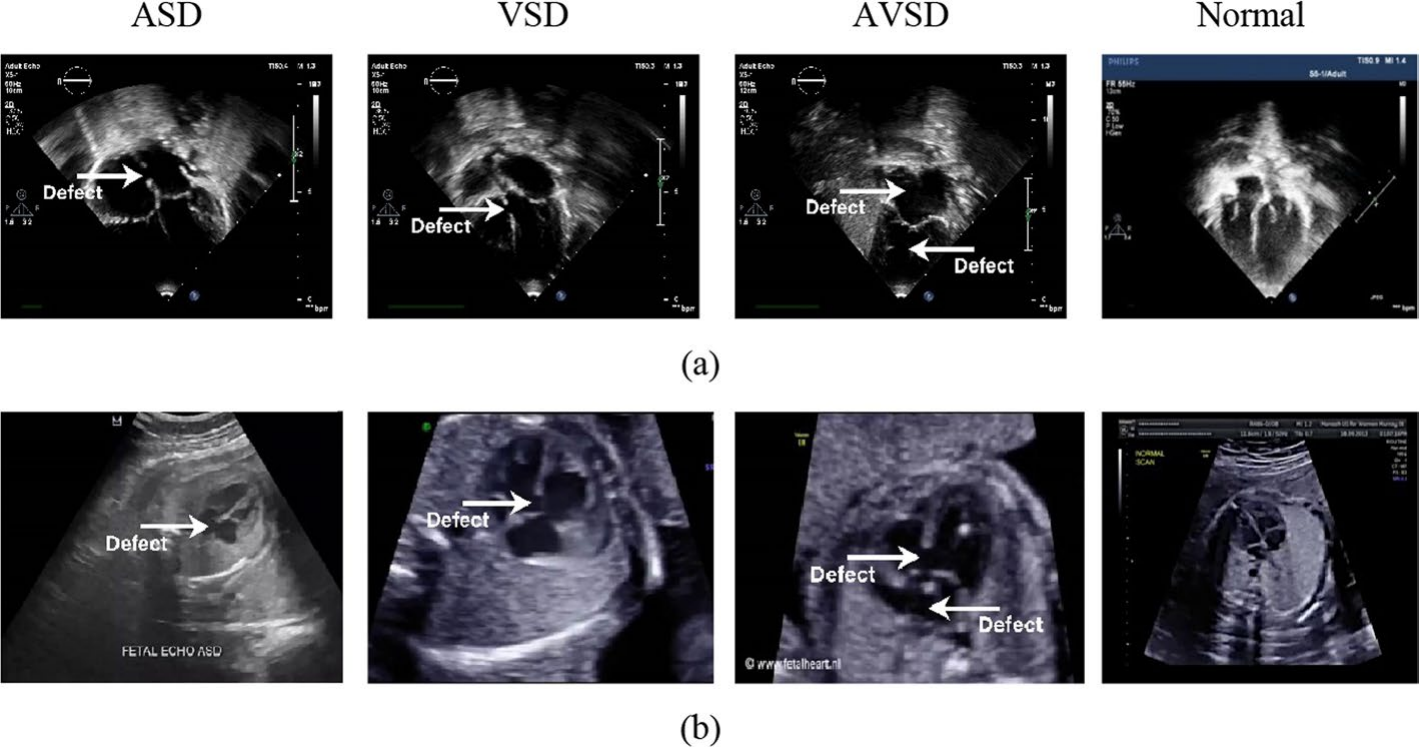
Example echocardiogram label of cardiac septal defect and normal heart. Images labeling only classifies cardiac images according to the type of anomalies and normal. This process is carried out after the segmentation process, by looking at the position of the defect from the segmentation results. The labelling process performed manually by the fetomaternal and pediatric cardiologist. The white arrow indicates the position of the defect. **a** Postnatal echocardiograms. **b** Prenatal echocardiograms

Cardiac septal defects without segmentation are not always straightforward to detect, however using only segmentation cannot produce defect interpretation. This study use the output from segmentation to achieve the decision of septal defect type with classification process (Fig. 2). The image labelling for defect classification is divided into two scenarios, (1) video recording of echocardiogram examination for postnatal hearts label is performed with apical 4-chamber view, apical 5-chamber view, parasternal long axis view, parasternal short axis view, and subcostal view. and (2) video recording of echocardiogram examination for prenatal hearts label is performed only with apical 4-chamber view, due to such view is an important and routinely performed view in fetal echocardiography as well as on a standard second trimester anatomy scan.

### Proposed a stacked residual-dense network model

**Fig. 3:**
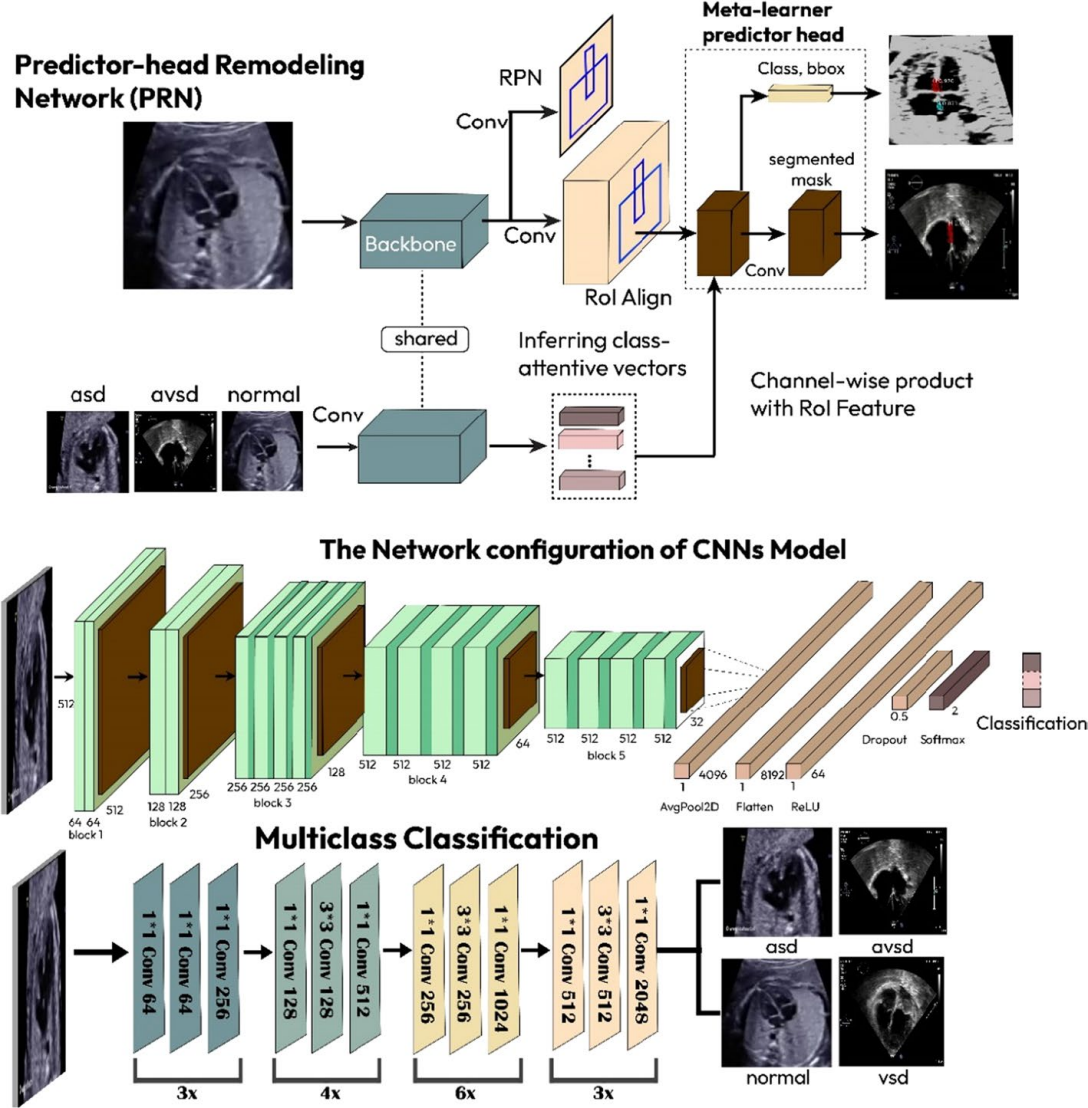
A stacked residual-dense network model based on two modalities echocardiography for automatic interpretation

The proposed workflow of a stacked residual-dense network model is divided into three main processes (Fig. 3) including, cardiac chamber and defect segmentation, defect classification, and decision. Two types of prenatal and postnatal echocardiogram data were used as inputs for the segmentation process (Table 1). Such cardiac echocardiograms have been annotated in the cardiac septum and defects (Fig. 1). Our stacked residual-dense network model uses an instance segmentation approach (Fig. 3). This approach was selected because the result is more detailed than semantic segmentation in visually expressing the segmented object [14, 19].

The segmentation involves two stages; the first stage involves a feature extraction backbone called region proposal network (RPN), with two network heads (i.e., bounding box object detection and mask segmentation heads), to predict the object class and regresses the final bounding boxes for each proposal [23]. The second stage is the segmentation head, called the fully connected network (FCN), which provides object masks for each object class and bounding box. The segmentation was developed on the basis of two CNN architectures, ResNet 50 and ResNet 101, with an intersection over union (IoU) threshold of approximately 50% (0.5) and a non-maximum suppression threshold of approximately 70% (0.7), to ensure good segmentation results.

The output from the segmentation process is the cardiac septum and the defect. Whole echocardiograms produced through segmentation are labeled according to the defect position and become the input in the classification process. The classifier is designed to predict three cardiac defects and a normal echocardiogram to determine whether the condition is ASD, VSD, AVSD, or normal. The multiclass classification process uses eight CNNs architectures, and the best performance is selected as the proposed model. The output of the proposed model is a complete interpretation of a cardiac septal defect.

The stacked residual-dense network model was implemented using the PyTorch 1.7.1 library and trained using a computer with the following specifications: an Intel Core i9-9920X CPU processor at 3.50 GHz, 490,191 MB RAM, GeForce 2080 RTX Ti by NVIDIA Corporation GV102 (rev a1), and an Ubuntu 18.04.5 LTS operating system.

### Model evaluation

Evaluation of our proposed a stacked residual-dense network model was based on six metrics for segmentation process: loss in classification, loss in segmentation, loss in detection, overlap between the input annotated (ground truth) and input predicted of each class in the IoU, overlap between the image annotated and image predicted of each class in the dice similarity coefficient (DSC), and overlap between the region of interest (RoI) annotated and RoI predicted for each bounding box in mean average mean precision (mAP) [14, 16, 23]. The performance of classification process was measured using the accuracy, sensitivity, and specificity metrics [13]. To assess the generalizability of the proposed model, we evaluated the overall performance degradation against the validation and unseen data.

## Results

In this section, we comprehensively analyze the experimental results of the prenatal and postnatal echocardiograms. The main difficulty of our learning process is the use of different cardiac standard planes by gynecologist fetomaternal and pediatric cardiology experts for prenatal and postnatal diagnosis when deciding the cardiac septal defect condition. The analysis is presented in the following section.

### Segmentation performance

In the segmentation process, Residual Network (ResNet) 50 and 101 architecture were compared as the backbones of the RPN. The IoU threshold was set to 50% (0.5) to ensure good segmentation prediction. In our experiment, the three types of cardiac septal defects, ASD, VSD, and AVSD, produced the IoU values exceeding 50% (0.5) based on the validation data (Table 2). This implies that the two models successfully recognized and segmented the cardiac defects in the atria and ventricle. We conducted an experiment with unseen data to ensure that the proposed model produced a robust network. Unseen clinical data refer to real-life conditions that typically differ from those encountered during training. However, it observe a slight decrease in performance compared to the predetermined IoU and DSC metrics, particularly for conditions such as ASD and VSD. The IoU value reaches under 50% (0.5) for ResNet 50 and ResNet 101, because, in normal conditions, prenatal and postnatal echocardiograms differ greatly in size and shape. The shape of the heart is fixed postnatally but varies greatly prenatally depending on the position of the fetus in the womb. In addition, opening and closing the valve in the prenatal heart changes its shape. Therefore, we selected ResNet 101, which has a higher IoU value than the ResNet 50 architecture.

**Table 2:** Instance segmentation performance with two backbones: ResNet50 and ResNet101 architectures

Condition	Validation data		Unseen data	
	ResNet50	ResNet101	ResNet50	ResNet101
*IoU (%)*				
ASD	55.69	57.07	26.96	41.21
AVSD	65.23	60.38	16.75	52.08
VSD	53.90	57.07	22.43	33.89
*DSC (%)*				
ASD	72.52	77.03	40.84	49.12
AVSD	75.82	74.39	44.20	73.97
VSD	75.17	75.84	46.84	44.06
*mAP (%)*				
Average	73.92	76.36	31.11	51.04

Our instance segmentation model uses non-maximum suppression with a confidence threshold of 70% (0.7), that is, any overlapping object with a confidence level below the threshold will be removed. Moreover, DSC and mAP should reach values greater than 70% to recognize and segment the object precisely. As shown in Table 2, the values of the two metrics obtained were over 70% in the validation data for both the ResNet 101 and ResNet 50 architecture. However, when tested by unseen data, 55.72% average DSC and 51.04% mAP using the ResNet 101 backbone, and 43.96% average DSC and 31.11% mAP using the ResNet 50 backbone were reached. From the metrics IoU, average DSC, and mAP, our model performance with ResNet 101 obtained a satisfactory result. Overlapping between the ground truth and predicted echocardiographic results was > 50%. This overlapping area was sufficient to recognize a defect in the septum. Therefore, our CNNs model applied to unseen data produced successfully segmented and well-recognized septal defects (Table 2).

**Fig. 4:**
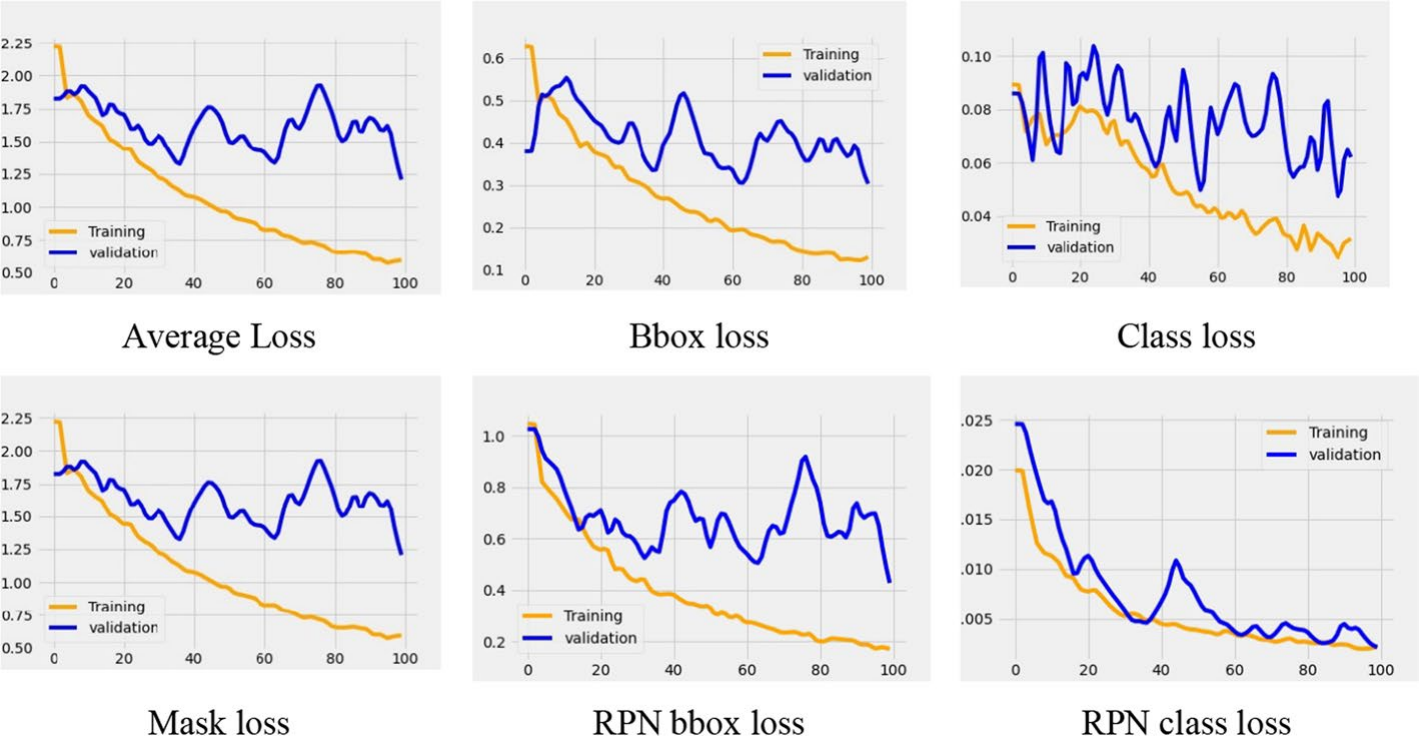
Instance segmentation loss with the ResNet 101 backbone in the RPNs

Instance segmentation using ResNet 101 can perform three processes simultaneously (i.e., classification, detection, and segmentation) in the RPN and FCN modules. Therefore, three losses can be generated: object detection loss as bounding box (bbox) loss, classification loss as class loss, and segmentation loss as mask loss. We observed that all the loss curves in the RPNs and FCNs during the training and validation processes decreased to the stability point, and the gap between the two curves was relatively small (Fig. 4). No overfitting condition was observed on the entire loss curve; the beginning of the curve tended to show a high validation loss, gradually decreasing to zero. The results indicate that adding more epochs can improve the model performance on the validation data. The segmentation model differs from most prior systems, which depend on mask prediction for classification. As the segmentation model attempts to learn a mask for each class, the classes do not compete in generating masks. Hence, the proposed instance segmentation model did not experience overfitting during the training process. Although the response fluctuated, it converged to various loss functions.

### Classification performance

**Fig. 5:**
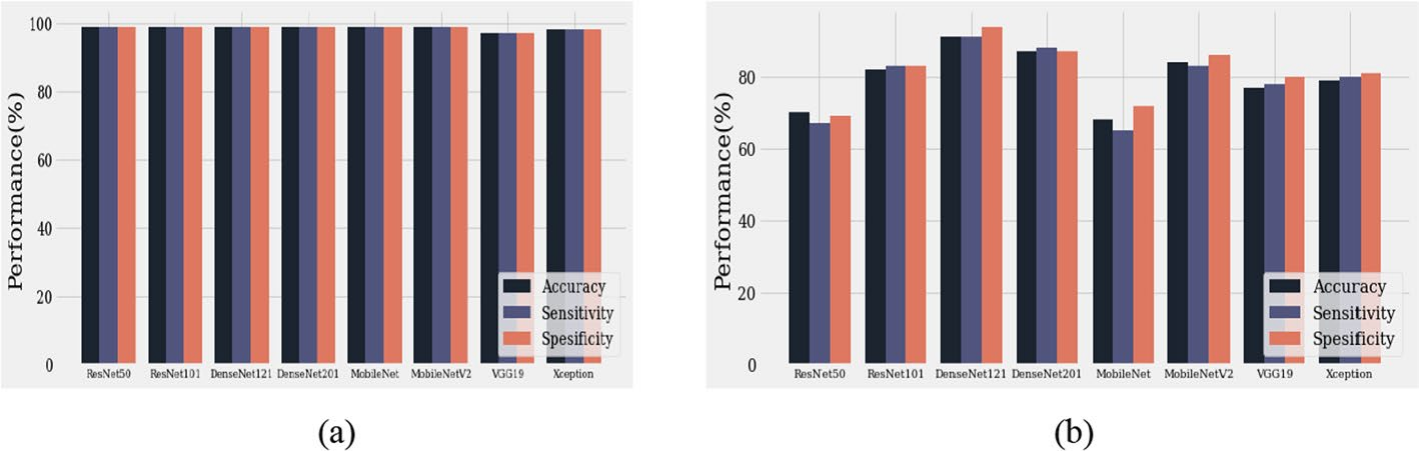
Classifier performance average. **a** Validation data performance. **b** Unseen data performance

**Fig. 6:**
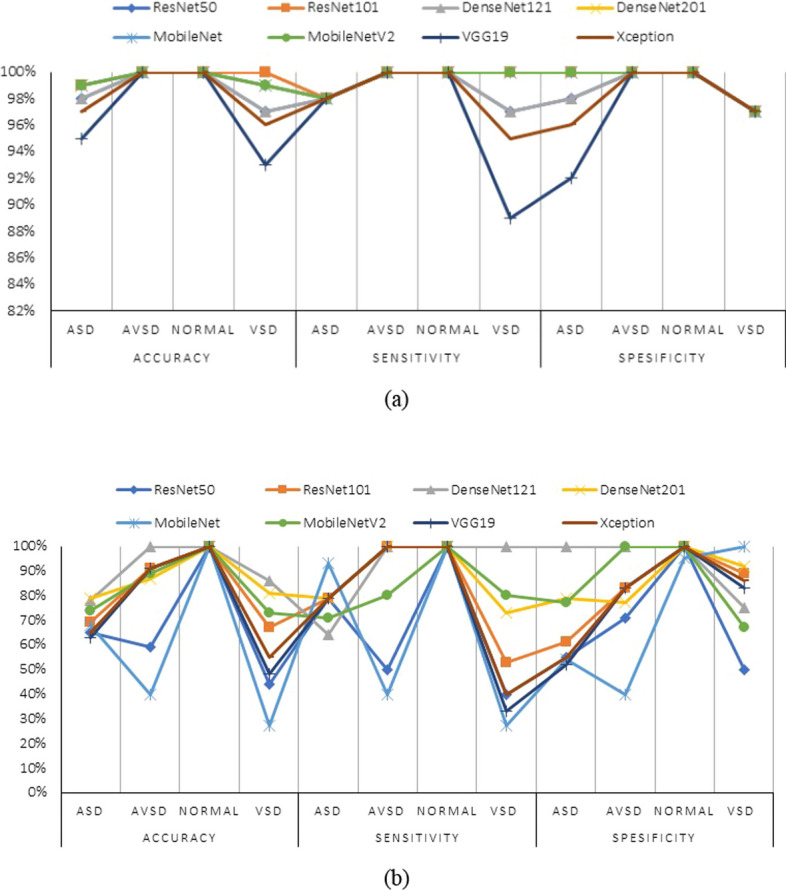
Classifier performance for each class. **a** Validation data. **b** Unseen data

Generalization demonstrates how well a trained model classifies and predicts unseen data, and a generalization model is important for medical applications. In many cases, a trained system fails to accurately predict unseen data. Therefore, data diversity is an essential factor for accurate prediction. Note that data diversity is not the only point to address when producing a generalized model and that poor hyperparameter configuration can also affect the prediction results. To address this issue, we experimented with two datasets, validation and unseen, using two RPN backbones. Figure 5 shows that our proposed model with DenseNet 121 outperformed the other architectures, with a prediction rate of 91% sensitivity, 94% specificity, and 91% accuracy using unseen data. In particular, for normal conditions with unseen data, we achieved a 100% negative predictive value in distinguishing normal from abnormal echocardiograms (Fig. 6). The VSD, AVSD, and normal class prediction rates were > 75%, and only the sensitivity of ASD was < 70%. Based on the overall prediction rate in the validation and unseen data for the eight classifier architectures, we can conclude that our proposed stacked architecture model with the ResNet 101 and DenseNet 121 architectures as the segmentation and classifier backbones, respectively, achieved satisfactory performance in both validation and unseen data.

**Fig. 7:**
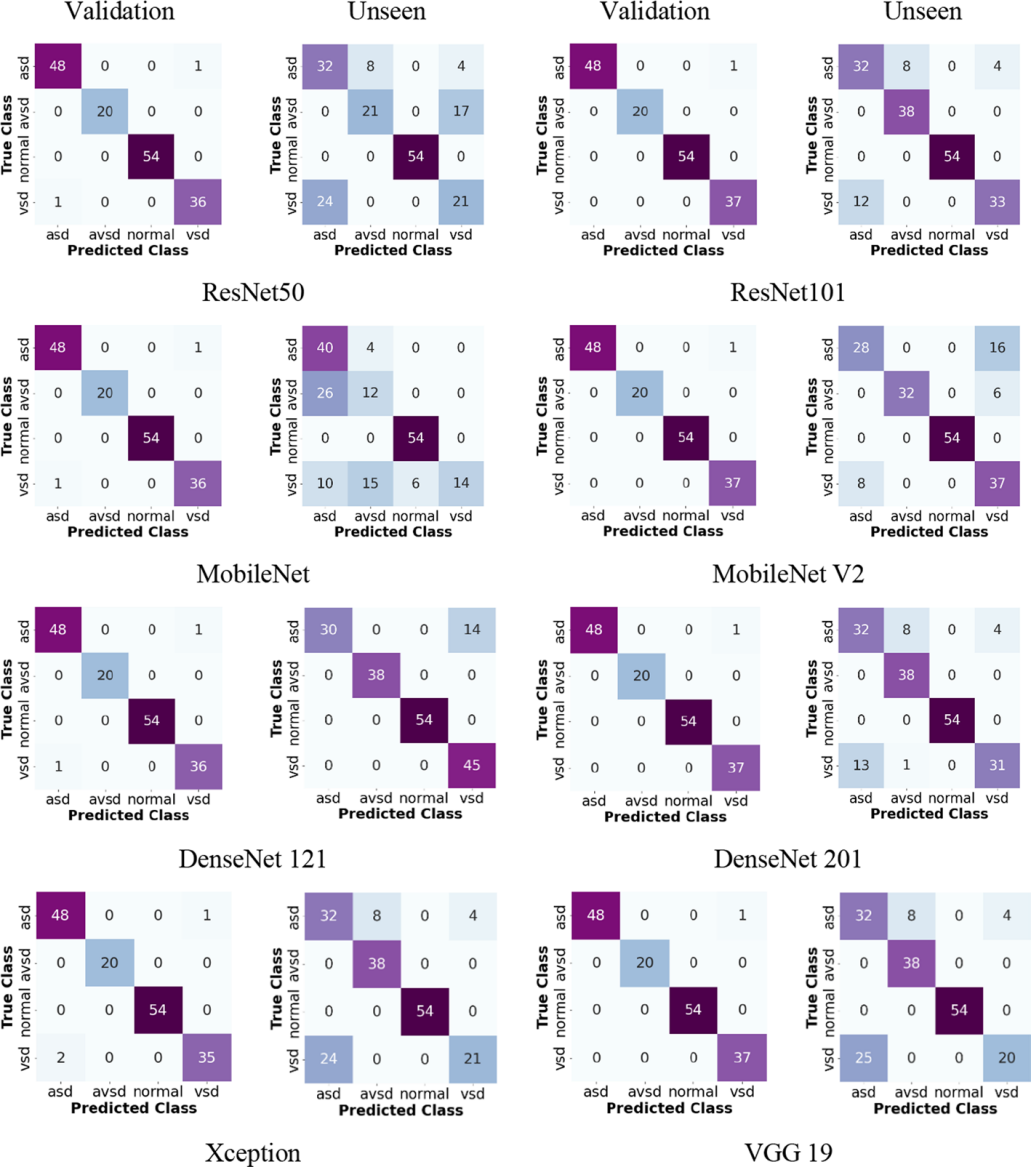
Confusion matrix with validation and unseen data for eight backbone architectures as classifiers

The performance results based on the validation and unseen data are presented as a confusion matrix and response curve. A confusion matrix was used to assess the performance of the classification model (Fig. 7). The confusion matrix shows that our stacked model achieves good performance. Eight types of CNN backbones, namely ResNet 50, ResNet 101, DenseNet 121, DenseNet 201, MobileNet, MobileNet V2, VGG 19, and Xception architectures, were compared to select the best performance. The DenseNet 121 performance metrics exceeded those of the other architectures, and this model produced a high positive predictive value, incorrectly predicting only five images. In this study, we present a straightforward and effective method for detecting, segmenting, and generating segmentation masks for cardiac septal defects on prenatal and postnatal echocardiograms. In general, the proposed model achieved the best performance with a stacked ResNet 101 and DenseNet 121 architectures.

### Proposed model evaluation

**Fig. 8:**
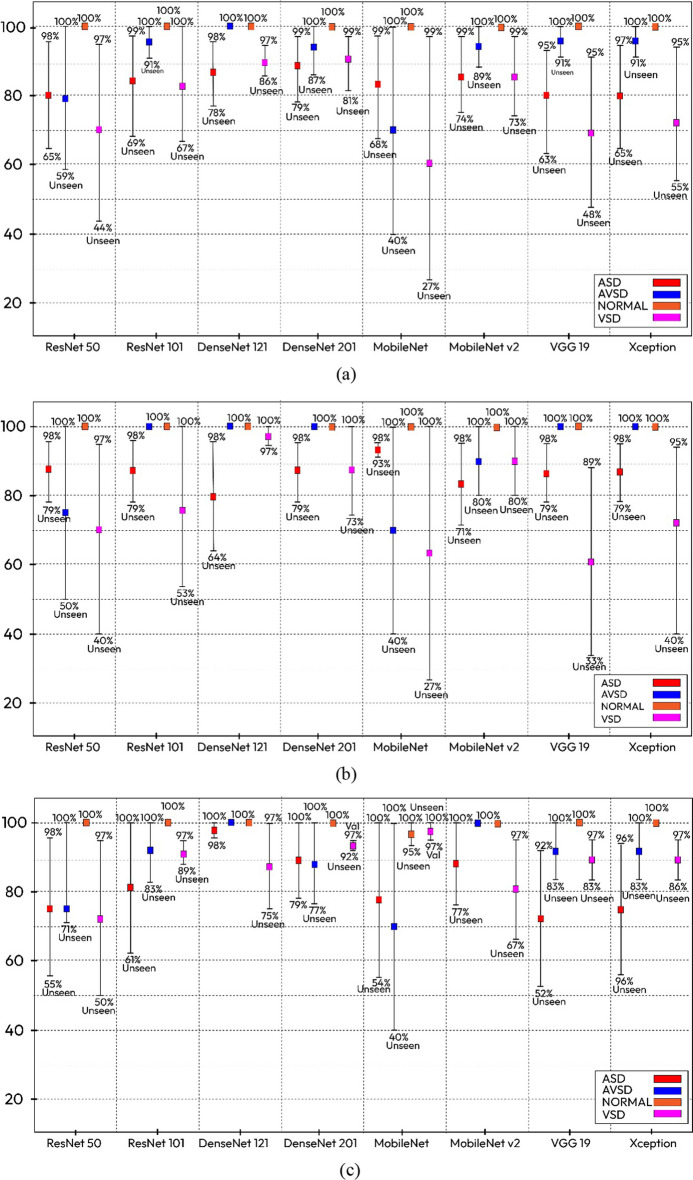
Variability performance between the validation and unseen data. **a** Accuracy. **b** Sensitivity. **c** Specificity

Model validation is the most important step in developing a model with excellent results. Overfitting is a problem that often occurs when a trained model performs exceptionally well on the samples used for training, but performs poorly on new unknown (unseen) samples, that is, the model does not generalize well. In general, a good performance prediction of the trained and optimized models on unseen samples is crucial to assess their generalization performance. Our proposed model with stacked architecture achieved a low bias value between the validation and unseen data. Figure 8a–c are illustrative summaries of the performance of all architectures in terms of accuracy, sensitivity, and specificity based on validation and unseen data. The DenseNet 121 architecture produced a low bias in its overall performance, compared with the other models in the ASD, VSD, and AVSD conditions, and generalized well to previously unseen objects. In two cases, an AVSD and a normal case achieved satisfactory segmentation and classification results on both validation and unseen data, with 0% variability or 100% prediction rate in the two datasets.

**Fig. 9:**
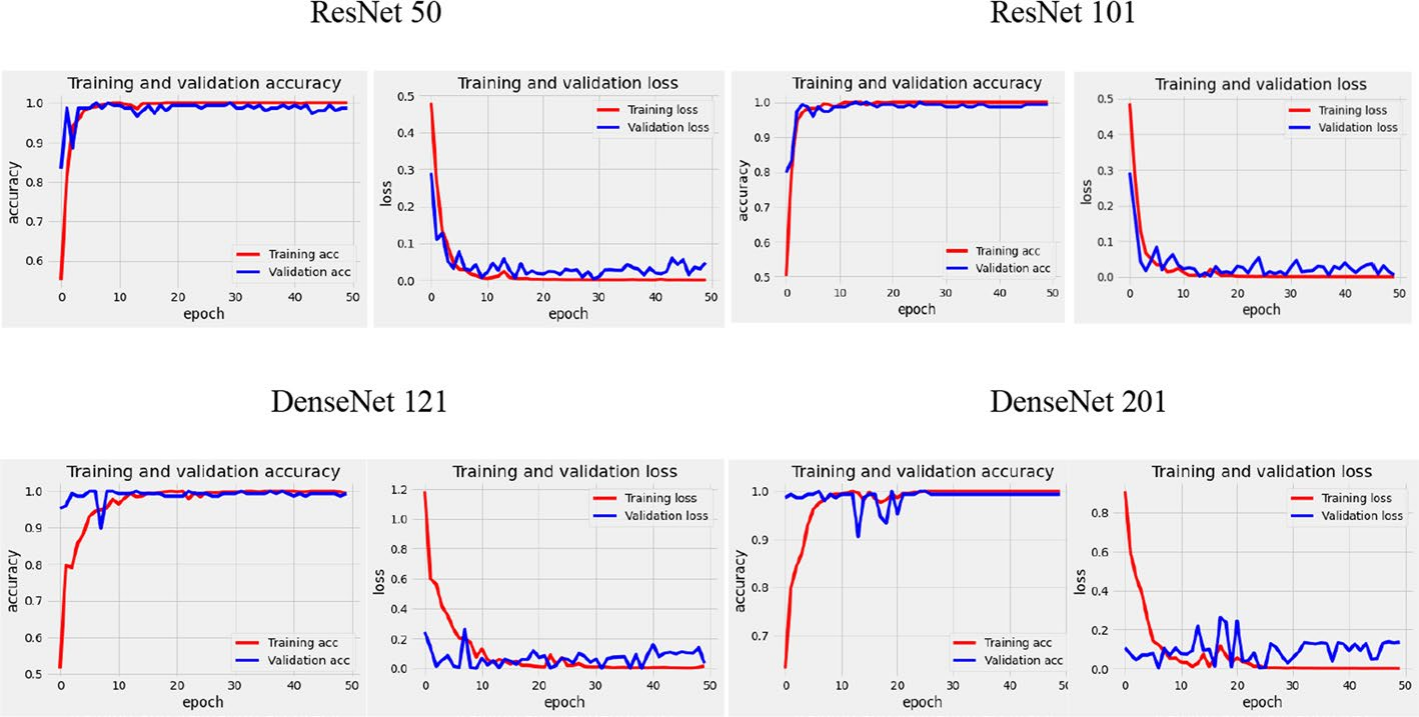
Sample performance accuracy and loss curve of four classifiers

As shown in Fig. 9, the accuracy and loss curves of the proposed model in the training and validation processes tended to be toward zero, and the accuracy curve gradually tended to be toward 100%. The training and validation processes responded significantly and steadily. Thus, the instance segmentation approach can be used in the validation process yielding results of approximately 99% accuracy, 99% sensitivity, and 99% specificity. However, in the testing process with unseen data, the prediction results decreased, but not significantly, and the overall performance still achieved > 90% accuracy, specificity, and sensitivity. As can be seen in Fig. 7, there are several misclassifications in ASD and VSD cases. This happens because when determining the defect, the position of the hole (whether it is in the atrial or ventricular chamber) is not considered. However, it is still implied that the algorithm had high performance with unseen data. The success of the proposed method is evident by its ability to automatically learn specific task feature representations. The proposed method can also be quickly integrated into healthcare facilities owing to its fast prediction capability.

**Fig. 10:**
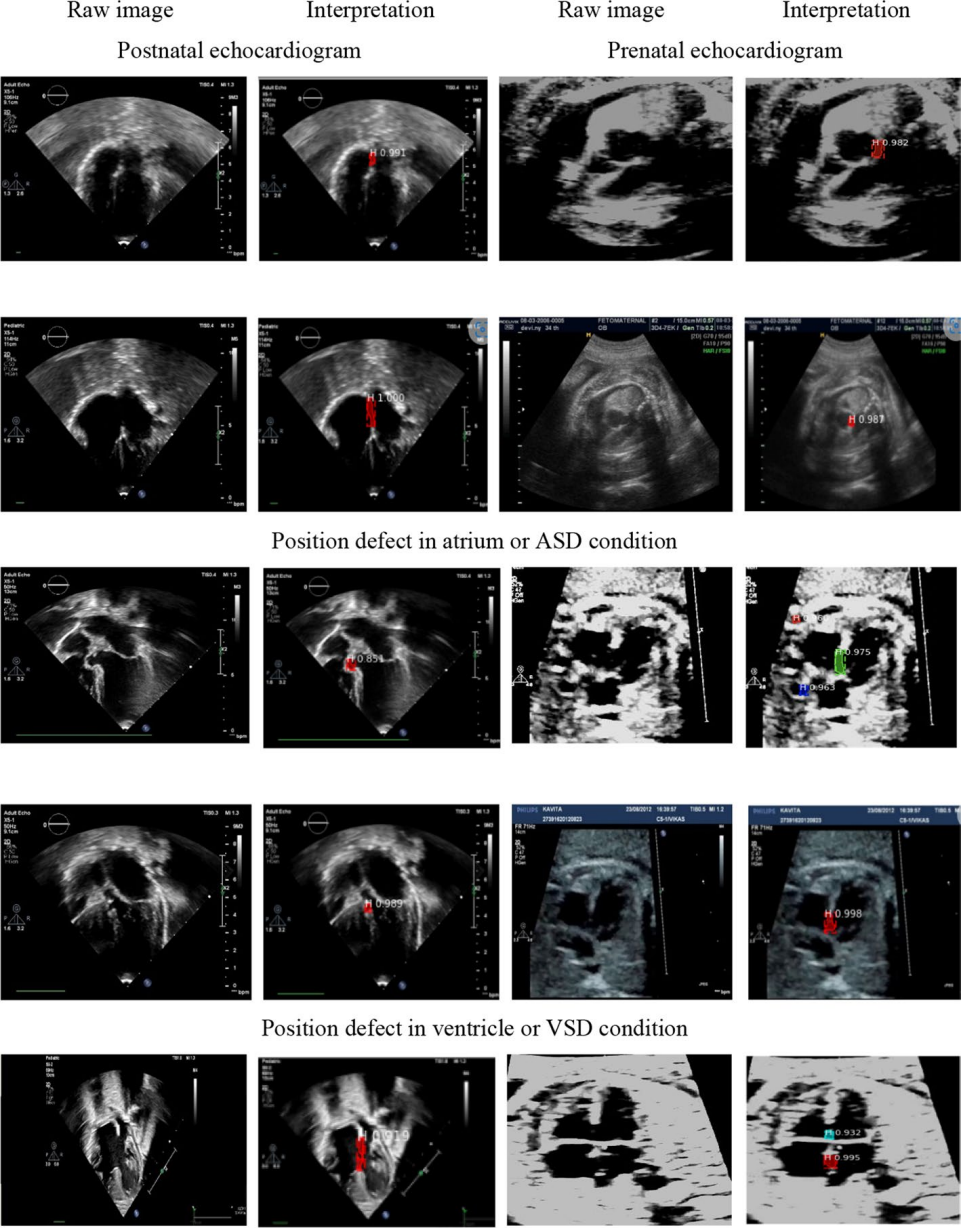

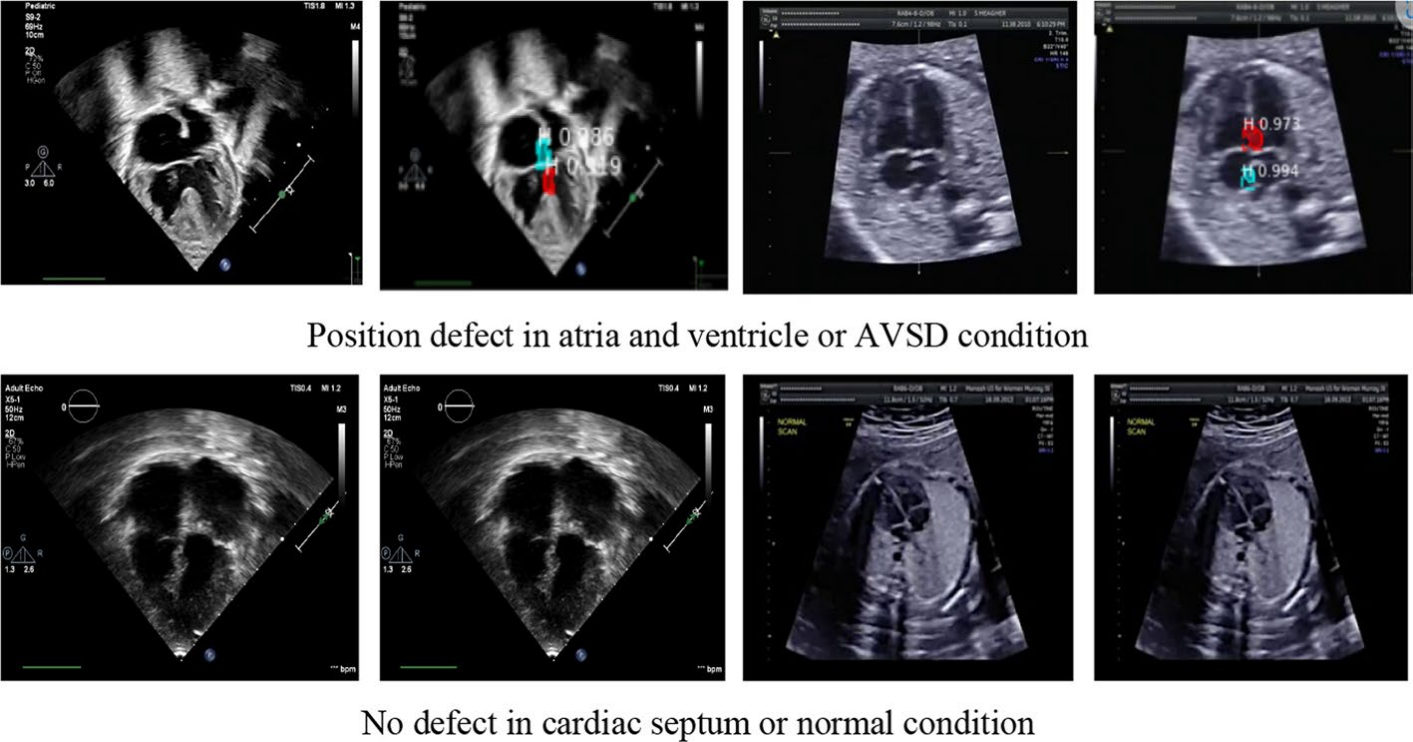
Cardiac septal defect classification based on prenatal and postnatal echocardiograms. The red area in the wall chamber is the defect with confidence value from the instance segmentation process

Our proposed model performed echocardiogram segmentation and distinguished pixel-level recognition and pixel-wise classification of the same echocardiogram with excellent accuracy. Figure 10 provides the visualization of the classification result, where the red area is the septal defect in the atria and ventricles. We demonstrated the model evaluation performance by incorporating two modalities because ultrasonography for prenatal and postnatal periods is different. To diagnose cardiac septal defect conditions prenatally, only the 4-chamber plane is required for ASD, VSD, and AVSD. However, 4-chamber, 5-chamber, and subcoastal planes should be used to identify a cardiac septal defect postnatally. Thus, the learning process for postnatally interpreting cardiac septal defects is more complicated. This case can be overcome using the proposed a stacked residual-dense network model because the process uses an adaption learning approach with three simultaneous stages in the architecture. This model can be used in echocardiogram areas without considering the shape and quality, regardless of blurred boundaries and subject-to-subject variations of echocardiograms.

Prenatal echocardiography is challenging because of the small size of the heart, imaging artifacts, and speckle noise [6, 8]. In addition, fetal movements cause the shape of the cardiac structure to vary for each patient, which is very different from the postnatal cardiac structure. Thus, predicting cardiac septal defects by incorporating the two cardiac shapes, prenatal and postnatal, is quite challenging. Hence, to prove the rationality of our stacked model, we compared the performance of our architecture against several state-of-the-art methods [13, 15, 23].

We are the first to conduct an experiment that incorporates prenatal and postnatal echocardiograms with outstanding results and detailed metrics. Table 3 indicates that our stacked model achieved the best performance in interpreting cardiac septal defects, with approximately 99% accuracy, 99% sensitivity, and 99% specificity. In addition, our model specifically predicted cardiac septal defects with three classes (i.e., ASD, VSD, and AVSD) and a normal class, whereas the previous work only predicted two classes (i.e., normal and diseases) [15, 24], and two classes (i.e., normal and TOF; normal and HLHS) [23]. Hence, our simple yet effective stacked instance segmentation and classification model with high accuracy is more reasonable than other models.

**Table 3:** Benchmarking with state-of-the-art CNN classifier models

Algorithm	Period	Number of class abnormality	Performance (%)		
			Accuracy	Sensitivity	Specificity
Residual learning [15]	Prenatal	2 classes (normal vs diseased) validation data	93	93	–
		2 classes (normal vs diseased) unseen data	91	91	–
Deep learning model [23]	Prenatal	2 classes (normal vs TOF validation data	–	75	76
		2 classes (normal vs HLHS) validation data	–	100	90
DGACNN [24]	Prenatal	2 classes (normal and diseased) validation data	85	–	–
Proposed Stacked model	Incorporating prenatal and postnatal	4 classes (normal, ASD, VSD, AVSD) validation data	99	99	99
		4 classes (normal, ASD, VSD, AVSD) unseen data	92	92	94

To further evaluate the effectiveness of our proposed model, we compared its performance with that of the model used by Qiao et al. on unseen data [15]. Our proposed model achieved 91% accuracy, 91% sensitivity, and 94% specificity for the four classes, whereas the RLDS achieved 91% accuracy and 91% sensitivity for the two classes. Therefore, our proposed model has a relatively higher accuracy in validation and unseen data and is thus credible enough for the initial interpretation of cardiac septal defects. Moreover, with 91% sensitivity and 94% specificity, our proposed model only produces seven false negative result. Moreover, the proposed architecture was able to detect normal cases perfectly even in unseen data. In addition, with the low number of false negatives it gives a relatively high sensitivity indicating that the model is able to detect a suspect patient correctly.

To benchmark the model that we are proposing whether it has expert-equivalent performance, we make a comparison between model and expert performance through 2D prenatal and postnatal US images. We invited two fetal-cardiologists and three pediatric-cardiologists to view US images one by one without echo effect. Every expert was given the same set as given in model evaluation. The test set for fetal-cardiologist consist of 1609 images with resolution of 800 × 600, on the other hand, the pediatric-cardiologist test 430 images with the same resolution of previous test set. The model prediction and human expert diagnosis is evaluate based on pre-defined ground truth and then compared using Kappa test. Based on the Kappa test, it was obtained that the Kappa value between the proposed model and expert 1 reach 0.912, it was considered that the model had almost the same ability as a fetomaternal expert to interpret cardiac septal defect condition in the fetus. However, expert 2 produces a lower Kappa value about 0.540 due to the human factor. On average, these results hold promise of increasing suitability in clinical practice as a supporting diagnostic tool for establishing the diagnosis (Table 4).

**Table 4:** Kappa value between proposed AI model and expert prediction with fetal’s heart and children’s heart US images

			Actual condition		Kappa value
			Cardiac septal defect	Normal	
Fetomaternal cardiologist prediction	Expert 1	Cardiac septal defect	765 (99.73%)	2 (0.27%)	0.912
		Normal	69 (8.26%)	766 (91.74%)	
	Expert 2	Cardiac septal defect	709 (69.10%)	317 (30.10%)	0.540
		Normal	59 (10.12%)	524 (89.88%)	
Pediatric cardiologist prediction	Expert 1	Cardiac septal defect	171 (83.01%)	35 (16.99%)	0.605
		Normal	50 (22.32%)	174 (77.68%)	
	Expert 2	Cardiac septal defect	101 (74.81%)	34 (25.19%)	0.291
		Normal	120 (40.68%)	175 (59.32%)	
	Expert 3	Cardiac septal defect	167 (71.06%)	68 (28.94%)	0.431
		Normal	54 (27.69%)	141 (72.31%)	

Whereas in the case of interpretation of a child's heart by a pediatric cardiologist, the results obtained when making predictions with US 2D images are, the expert 1 produces strong concordance level with a Kappa value of 0.605, expert 2 produce low concordance level with a Kappa value of 0.291, and expert 3 reach a moderate concordance level with a Kappa value of 0.431.

This experiment is almost similar to the study conducted by Madani et al. [13] which compared the accuracy of AI models with certified echocardiographers for classifying 15 standard echocardiogram views. In such model, it was found that the accuracy of the AI model was 97.8% above the accuracy of a certified echocardiographer, who only had a 70.2–84% accuracy rate for classifying a standard echocardiogram view. It can be concluded that the suitability of the interpretation of cardiac septum defects between the AI model and the interpretation by a cardiac consultant varies at low, medium, and strong levels of concordance.

The various results of the Kappa test are related to the accuracy of the interpretation which also varies based on two fetomaternal cardiac consultants and three pediatric cardiac consultants (Table 4). This is in accordance with the research of Anderson et al. [9] which identified differences in the interpretation of echocardiogram examinations between cardiac consultants (experts) and also between beginners (trainees). Consultant pediatricians and fetomaternal usually assess whether there is a cardiac septal defect or a normal heart, not just based on the 2D-images. This is especially true for small defects which are sometimes difficult to detect if you only look at the image.

## Discussion

The clear benefit to early diagnosis and treatment of cardiac septal defect made the need for accurate, scalable screening for cardiac septal defect is going stronger, while sensitivity and specificity for cardiac septal defect detection are quite variable at centres and clinics worldwide and in many centres remain quite low [25]. To address this, we investigated the impact of incorporating real-world prenatal and postnatal ultrasounds with cutting-edge DL to achieve expert-level cardiac septal defect detection from difficult diagnostic challenges in ultrasound. Our approach to both model design and testing ensured interpretability at several levels, which can help with clinical adoption. The DL model’s performance and speed allow its integration into clinical practice as software onboard ultrasound machines to improve real-time acquisition and to facilitate telehealth approaches to prenatal and postnatal care [26, 27].

The segmentation algorithm was evaluated regarding IoU and DSC. IoU refers to the area of the intersection of the predicted boundary and the actual boundary compared to the area of the union. The higher the IoU, the more accurate the target segmentation result. Moreover, the shape of the heart is mostly variable and irregular which will affect the size of the defect. Therefore, the segmentation effect of the heart defect was evaluated regarding the DSC. Such value refers to a measure of the overlap between the segmentation result and the standard area (marked by expert). However, there is no gold standard that states how much IoU and DSC values are used for medical imaging cases. To ensure good performance, at least half of the prediction areas overlap with the ground truth (IoU >  = 0.5) to produce a high confidence value as well as DSC. In our model by using training data, we reach IoU and DSC over 50%. However, the IoU/DSC value slightly decreased from 0.5, when it testing use testing data (Table 2). Such data is taken from new patient who are not in the training data. It mean our model is able to predict the defect in cardiac imaging for two echocardiographic modalities.

In the previous studies, several automatic classifications have been proposed to clearly prediction cardiac anatomical structures. However, in many cases, a trained system fails to accurately predict unseen data (testing data). Image segmentation is the process of automatic or semi-automatic detection of boundaries within a 2D image. The segmentation result can then be used to obtain further diagnostic insights. The combination between two process is conducted in this study to improve the classifier prediction rate. Due to, it is difficult to accurately identify the cardiac structure especially to predict the heart defect with small size, imaging artifacts, speckle noise, fetal rib shadowing, and missing boundaries [6, 7]. Our proposed model performed echocardiogram segmentation and classification with satisfactory performance, the all values reach over 90% in terms of accuracy, sensitivity and specificity by using unseen data (Table 3). As a result, it can help the clinician to detect cardiac septal defect at the early stage of gestation which increases the survival rate of new-borns with cardiac septal defect.

Our proposed model can explain the classifiers’ result by visualizing the segmentation results, unlike the conventional classifier model [16, 21, 23], which cannot visualize the selected results. A clear explanation to cardiologists while assisting them in medical diagnosis and knowledge of the defect features increases their understanding and confidence regarding the results obtained. We do not need visualization with a heatmap because the segmentation results show an area of defect in the cardiac septum before providing the final decision. For retrospectively collected images, the model could be used as standalone software for which a user uploads a study and receives model-chosen and diagnostic predictions. Finally, the automated cardiac septal defect diagnostic with incorporating two types of US has the potential to transform clinical practice in multiple ways, by nonexperts in primary care and rural settings.

Echocardiogram examination is a very important process in the management of patients with heart disease and what is currently being done is still manual so that the interpretation result of the examination is very dependent on the operator [9]. Even though the interpretation of the results of an echocardiogram examination performed by humans is subjective and influenced by thoughts and feelings so that interpretations can differ between experts [9, 13]. Our model is potentially helpful for medical staff untrained in cardiac imaging, despite the promising results, our study still has limitations as follow;

- Limited echocardiographic plane was used, and patient variation for achieving high sensitivity and accuracy in detecting cardiac septal defects remains lacking. Further research should improve the prediction rate for unseen data and expand this study to other abnormality conditions, which might contribute greatly to this research field. The variations in cardiac anatomical structures are very complex, to make a reliable and perfect model, more samples are needed and combined it with image enhancement model to increase the quality of US video.
- The model built in this study uses US images only to predict cardiac septal defects or normality, not to predict the size of the defect. In future research, the proposed model will be developed to be able to predict the type of cardiac septal defect based on the size of the defect, so that it can provide an interpretation of the defect size from small to large.
- The data acquisition process uses limited devices (GE Voluson E6 and Philips EPIQ 7C) and due to this method requiring on segmentation process to detect the heart defect, there is no use of color doppler as part of the training and testing process to identify heart defects.
- The data set uses postnatal imaging occurred in patients > 2 months of age, and prenatal imaging occurred in patients 22–28-week gestation. We do not perform postnatal imaging for new-born or immediately after birth.

## Conclusions

Early diagnosis of cardiac septal defects based on echocardiograms can be performed initially during the prenatal or postnatal period. However, prenatal cardiac septal defects are difficult to identify owing to several factors, including small size, artifacts and speckle noise, shadowing, and missing boundaries. Delayed identification of postnatal cardiac septal defects can occur because not all suspected cases are examined with echocardiography, even if such a process is the gold standard examination to diagnose the condition. In this study, we presented a straightforward and effective method for automatic segmentation and classification of prenatal and postnatal echocardiograms to generate an accurate interpretation regarding cardiac septal defects. The experiment was conducted using an end-to-end learning process with ResNet 101-based segmentation and DenseNet 121-based classification. The value of this study is best understood against the background of its limitations. The results generalize well to previously unseen objects in other patients. We observe that our results are an important as the initial step for highlight the possibility of adopting DL to provide a fully automated solution for interpreting echocardiograms, which can support clinicians and augment their clinical care.

## Declarations

### Availability of data and materials

The datasets generated and/or analyzed in this study are available in https://github.com/ISySRGg/Echo-Infant
